# Supplementary material for: The interactome of intact mitochondria by cross-linking mass spectrometry provides evidence for coexisting respiratory supercomplexes
Source: Mol Cell Proteomics. 2017 Dec 8;17(2):216–32. doi: 10.1074/mcp.RA117.000470 (PMC5795388; doi:10.1074/mcp.RA117.000470)
Supplement: Supplemental Data [file supp_RA117.000470_133922_0_supp_23978_fzffwf.zip › spectra_annotation/mito_spectra_annotation/216-1-4-1-10-1.pdf]

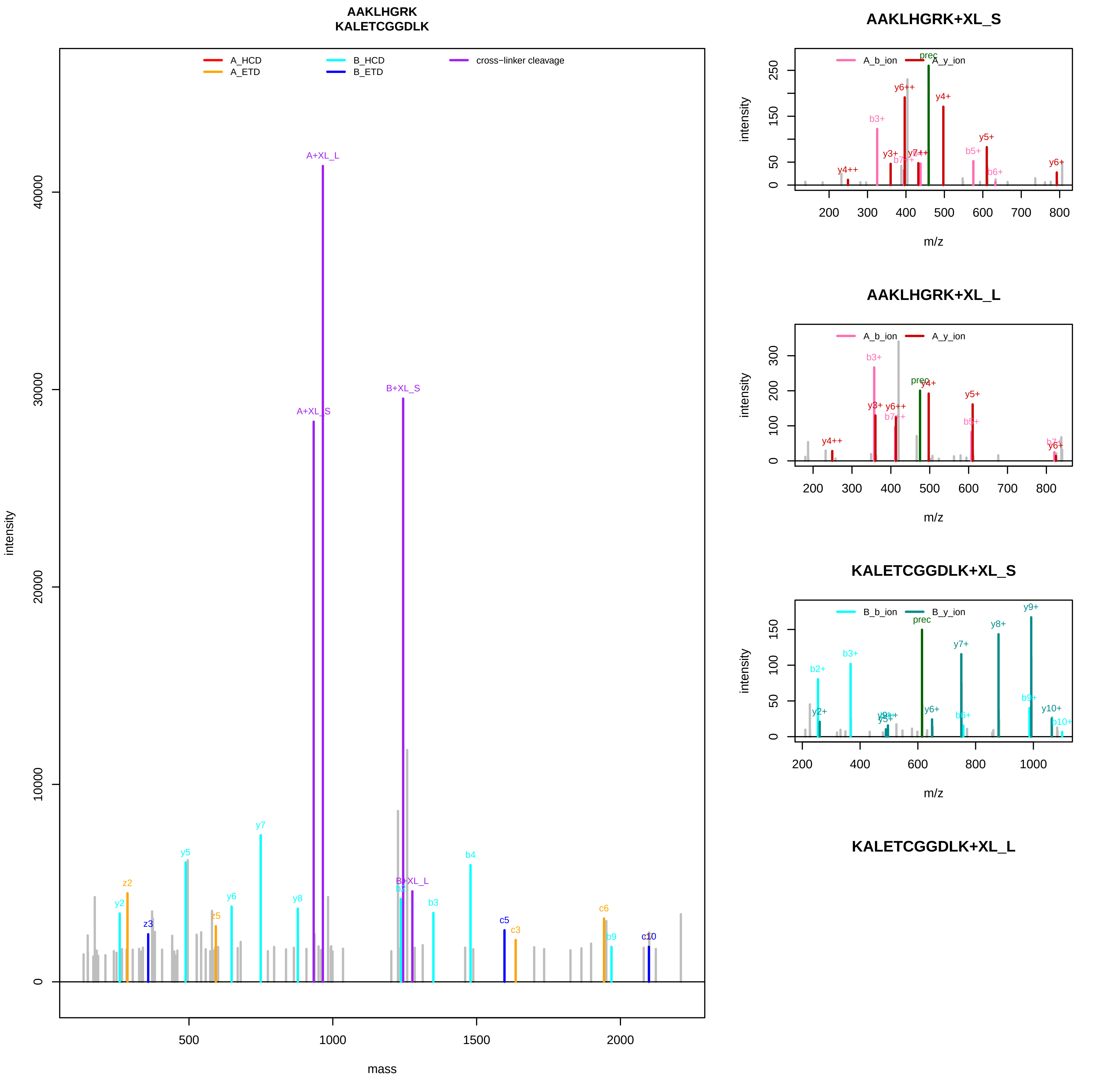
AAKLHGRK
KALETCGGDLK
A_HCD
A_ETD
B_HCD
B_ETD
cross−linker cleavage
A+XL_L
A+XL_S
B+XL_S
B+XL_L
y2
z2
z3
y5
z5
y6
y7
y8
b2
b3
b4
c5
c3
c6
b9
c10
intensity
mass
0
10000
20000
30000
40000
500
1000
1500
2000
AAKLHGRK+XL_S
A_b_ion
A_y_ion
prec
y6++
y4+
b3+
y3+
y7++
y5+
b5+
y4++
b6+
y6+
m/z
AAKLHGRK+XL_L
b3+
y3+
y6++
b7++
y4+
y5+
b5+
y4++
y6+
KALETCGGDLK+XL_S
B_b_ion
B_y_ion
y9+
y8+
y7+
b3+
b2+
y2+
y6+
b6+
b9+
y10+
b10+
KALETCGGDLK+XL_L
